# Comments on: “What Is Developmental Dyslexia?” *Brain Sci.* 2018, *8*, 26. The Relationship between Eye Movements and Reading Difficulties

**DOI:** 10.3390/brainsci8060100

**Published:** 2018-06-04

**Authors:** Hazel I. Blythe, Julie A. Kirkby, Simon P. Liversedge

**Affiliations:** 1 Psychology, University of Southampton, Southampton SO17 1BJ, UK; 2 Psychology, Bournemouth University, Fern Barrow, Poole BH12 5BB, UK; jkirkby@bournemouth.ac.uk; 3 School of Psychology, University of Central Lancashire, Fylde Rd, Preston PR1 2HE, UK; SPLiversedge@uclan.ac.uk

**Keywords:** eye movements, dyslexia, reading

## Abstract

We are writing in response to the review article: Stein. J. (2018). What is Developmental Dyslexia? *Brain Sciences*, *8*, 26, doi:10.3390/brainsci8020026. We consider that the section entitled, “Eye Movement Control”, presents a misleading characterisation of current empirical and theoretical understanding. We outline five specific points relating to Stein’s views on eye movement control and developmental dyslexia with which we disagree and conclude that disruption to oculomotor behaviour occurs as a consequence of processing difficulty that individuals with dyslexia experience as they engage in reading.

We are writing in response to the review article: Stein. J. (2018). What is Developmental Dyslexia? *Brain Sciences*, *8*, 26, doi:10.3390/brainsci8020026. In his article, Stein provides a position statement with respect to causative factors and the nature of dyslexia [1]. There is one section of the article in particular, Section 13, entitled “Eye Movement Control”, that we consider presents a misleading characterisation of current empirical and theoretical understanding. Here, we outline five specific points relating to Stein’s views on eye movement control and developmental dyslexia with which we disagree.

First, Stein’s consideration of the results of some eye movement studies is selective, leading to a representation of the findings that does not reflect the full pattern that was originally reported by the authors. For example, Adler-Grinberg and Stark reported that the eye movement data from their groups of children with and without dyslexia “could not be differentiated” (pp. 561 and 563) when analysing saccadic latency, accuracy, and velocity, as well as fixation location, duration, and accuracy on nonreading tasks [2]. Although differences in smooth pursuit eye movements are described, the only statement concerning formal statistical analysis of those differences is, “a significantly higher number of the dyslexic children displayed this behaviour” (p. 561). It is also noted that a number of children in the typically developing group also showed these “abnormalities” (p. 561) in smooth pursuit. These data indicate that, in contrast to the view expressed by Stein, on nonreading tasks without linguistic processing demands, there were very few group differences in eye movement behaviour. A range of formal, statistically significant differences between groups’ eye movement behaviour was reported, however, when the children performed a reading task. Here, children with dyslexia were reported to make more and longer fixations and had slower reading rates than typically developing children. In our view, these data strongly suggest that cognitive processing difficulty during the reading task resulted in different patterns of eye movement behaviour for the children with dyslexia. Indeed, we note that Adler-Grinberg and Stark’s data have been cited in other review articles which draw this conclusion (e.g., [3,4,5,6]). In his review, Stein simply states, “Slow motion processing in dyslexics therefore causes the eyes to fail to keep up with it, so their pursuit eye movements fall further and further behind, necessitating frequent catch up saccades”, (p. 5) and argues on the basis of these data (among others) that we must reconsider a causal role for atypical eye movement control in individuals with dyslexia. In our view, in order to fully understand the complex relationship between eye movement behaviour and reading difficulties, it is critical to simultaneously consider a large number of eye movement parameters, and more specifically, to consider how variability in those different parameters relate both to each other and to the visual and cognitive demands of the task employed. Selectively focusing on one of a number of different eye movement parameters will likely lead to erroneous conclusions.

Second, research investigating monocular and binocular aspects of eye movement control during reading are conflated in this section of the paper, resulting in a controversial and, we believe, unfounded conclusion. Stein cites four studies in Section 13 of his review, two of which focus upon aspects of binocular coordination [7,8]. He makes a series of assertions that associate atypical oculomotor behaviours with dyslexia, and a number of those statements pertain specifically to aspects of binocular coordination (for example, discussing “large vergence errors” and “inaccurate and divergent saccades”, p. 5). Stein concludes, “the consensus remains that any eye movement problems seen in dyslexics are the result, not the cause of reading problems. This needs to change,” (p. 5), making specific reference to Rayner’s work. Whilst seemingly consistent with the preceding arguments, this conclusion fails to note that Rayner’s work focuses on monocular eye movement behaviour during reading as an index of cognitive processing difficulty. In fact, across a number of independent publications, Rayner clearly and repeatedly argued that, in order to show that poor oculomotor control causes dyslexia, it would be necessary to demonstrate atypical patterns of eye movement behaviour on both reading and nonreading tasks (e.g., [3,4,5,6]). To reiterate, Rayner’s stance was based on the association between monocular patterns of eye movement behaviour during reading and nonreading tasks in relation to cognitive processing difficulty, and his views that “eye movement problems seen in dyslexics are the result, not the cause of reading problems” [1] (p. 5) were never based on data examining binocular coordination. The conflation of arguments that have been formed on the basis of data from theoretically distinct monocular experiments (e.g., [3,4,5,6]) and binocular experiments (e.g., [7,8]) undermines Stein’s argument. With respect to monocular measures of reading behaviour and reading-like behaviour for nonlinguistic stimuli, we argue that the body of published evidence strongly indicates that cognitive processing difficulty during reading causes disruption to patterns of eye movement behaviour (and not vice versa). Distinct from this, a number of studies have been reported in recent years that focus specifically on aspects of binocular coordination in individuals with dyslexia and we turn to this area of research in our next point of response.

Third, key publications providing solid empirical data that are problematic for the position advocated by Stein are omitted from his review of eye movement research investigating dyslexia (e.g., [9,10,11,12,13]). All of these papers examined aspects of binocular coordination in children in relation to reading ability. Bishop et al. found no differences in reading ability between children classed in terms of their reference (dominant) eye [9]. Furthermore, they found no difference between children classed as good or poor readers on acuity, convergence deficiency, stereopsis, phoria, squint, reference eye, and association between eye and hand reference. The authors stated, in relation to earlier reports from a different research group, “We failed to replicate their findings of a raised incidence of convergence deficiency, defective stereopsis and esophoria in children with specific reading disability”.

Cornelissen, Munro, Fowler, and Stein reported no differences in vergence behaviour during reading between typically developing children, children with reading difficulties who had a stable referent eye, and children with reading difficulties who did not have a stable referent eye [10]. This lack of differences was robust across two analyses, in which the groups were either matched for chronological age or for reading age. Lennerstrand et al. reported data from a large-scale longitudinal study comparing children with and without dyslexia [12,13]. Children with dyslexia were found to have lower visual acuity and contrast sensitivity than typically developing children, but there were no group differences in ocular dominance, phoria, or fusional range [12,13]. With respect to eye movements, analyses indicated poorer binocular coordination during reading in children with dyslexia compared to typically developing children, but there were no differences between the two groups on these measures during a nonreading assessment [12]. Consistent with this, Kirkby et al. found no differences in binocular coordination between children with or without dyslexia on a nonreading task that was designed to mimic the oculomotor demands of sentence reading but without imposing any linguistic processing demands [11]. In contrast, differences between these two groups’ binocular coordination were found during a sentence reading task. Considering the results across these studies, the view that atypical binocular coordination may cause the reading difficulties associated with dyslexia is not well supported. Clearly, this perspective is at odds with Stein’s position.

These papers focus on aspects of binocular coordination which, as we have argued above, is distinct from the role or roles that monocular aspects of oculomotor control have been posited to play in dyslexia. For brevity’s sake, within monocular measures, we provide here one example to support our argument. In his review, as discussed above, Stein focuses on smooth pursuit movements within the numerous analyses reported by Adler-Grinberg and Stark [2], noting that differences were observed between the groups of children with or without dyslexia. Whilst this is, indeed, what the authors originally reported, Stein’s review does not include the studies that have failed to find such differences (e.g., [14,15,16]). Considering just one of these studies, Judge, Caravolas, and Knox found that phonological processing, but not smooth pursuit, strongly correlated with nonword- and word-decoding in readers with dyslexia [15]. They concluded, “no evidence emerged to suggest that poorer smooth pursuit performance … led to poorer literacy” (p. 1185). For a wider review of studies that have examined the role of oculomotor behaviour in dyslexia, see [17]. With respect to Stein’s review, we suggest that consideration of the empirical context is less than comprehensive.

Fourth, in our view, in order to show that impaired magnocellular development is a primary cause of dyslexia, it would be necessary to compellingly and empirically demonstrate:

(a) That atypical magnocellular function results in atypical eye movement control within the population of individuals with dyslexia. The demonstration of co-occurrence between neurological differences and behaviour is, in itself, insufficient (e.g., [18]).
(b) Subsequently, that atypical eye movement control results in reading difficulties. To date, there are a number of publications in which the opposite conclusion is drawn, namely, that eye movement data indicate that cognitive processing difficulties during reading are the cause, and not the consequence, of eye movement control (e.g., [3,4,5,6,11,19]).

It is our contention that, as yet, there has been no such demonstration in the literature. We do not posit that such empirical evidence will not be reported in the future. We strongly argue, however, that the suggestion of a causal role for atypical eye movement control in reading difficulties, resulting from differences in magnocellular function, is not supported by the published literature to date.

Fifth, we note that, very often, studies that have shown atypical vergence behaviour in children with dyslexia have recruited child participant populations from specialist hospitals. Indeed, this is the standard approach Stein adopts in relation to his own experimental populations (e.g., [20,21,22,23]), and often those of Kapoula, Bucci, and colleagues whose work Stein cites (e.g., [7,8]). We contend that such participant populations are not representative of the more general population of individuals with dyslexia. The majority of children within the United Kingdom who are diagnosed with dyslexia will receive support and assessment from a range of specialists and services that may include teachers, educational psychologists, and support organisations such as Dyslexia Action or the British Dyslexia Association, but not typically from medical professionals, and they typically would not be assessed or supported within a hospital setting. It seems reasonable to posit, therefore, that children with reading difficulties who have been referred to a specialist eye unit within a hospital might in fact be a subset of children who have additional, comorbid orthoptic difficulties. Empirical demonstration of differences between such children, and the broader population with dyslexia, have not yet been reported as far as we are aware; that said, it is vital to consider the influence of a likely bias within a participant sample when interpreting the results from a study.

In summary, we disagreed with several of the views put forward by Stein in his original article. Our evaluation of the broader empirical literature leads us to an alternative conclusion; specifically, that disruption to oculomotor behaviour occurs as a consequence of processing difficulty that individuals with dyslexia experience as they engage in reading. It is for this reason that we felt compelled to write this response article.
